# Isometric exercises do not provide immediate pain relief in Achilles tendinopathy: A quasi‐randomized clinical trial

**DOI:** 10.1111/sms.13728

**Published:** 2020-06-14

**Authors:** Arco C. van der Vlist, Peter L. J. van Veldhoven, Robert F. van Oosterom, Jan A. N. Verhaar, Robert‐Jan de Vos

**Affiliations:** ^1^ Department of Orthopedic Surgery and Sports Medicine Erasmus MC University Medical Center Rotterdam the Netherlands; ^2^ Department of Sports Medicine Haaglanden Medical Center The Hague the Netherlands

**Keywords:** loading, pain management, rehabilitation, tendon

## Abstract

**Background:**

Isometric exercises may provide an immediate analgesic effect in patients with lower‐limb tendinopathy and have been proposed as initial treatment and for immediate pain relief. Current evidence is conflicting, and previous studies were small.

**Objective:**

To study whether isometric exercises result in an immediate analgesic effect in patients with chronic midportion Achilles tendinopathy.

**Methods:**

Patients with clinically diagnosed chronic midportion Achilles tendinopathy were quasi‐randomized to one of four arms: isometric calf‐muscle exercises (tiptoes), isometric calf‐muscle exercises (dorsiflexed ankle position), isotonic calf‐muscle exercises, or rest. The primary outcome was pain measured on a visual analogue scale (VAS) score (0‐100) during a functional task (10 unilateral hops) both before and after the intervention. Between‐group differences were analyzed using a generalized estimation equations model.

**Results:**

We included 91 patients. There was no significant reduction in pain on the 10 hop test after performing any of the four interventions: isometric (tiptoes) group 0.2, 95%CI −11.2 to 11.5; isometric (dorsiflexed) group −1.9, 95%CI −13.6 to 9.7; isotonic group 1.4, 95%CI −8.3 to 11.1; and rest group 7.2, 95%CI −2.4 to 16.7. There were also no between‐group differences after the interventions.

**Conclusion:**

The isometric exercises investigated in this study did not result in immediate analgesic benefit in patients with chronic midportion Achilles tendinopathy. We do not recommend isometric exercises if the aim is providing immediate pain relief. Future research should focus on the use of isometric or isotonic exercise therapy as initial treatment as all exercise protocols used in this study were well‐tolerated.

## 1 INTRODUCTION

Chronic tendon overuse injuries (tendinopathy) are common and account for 30%‐50% of all sports‐related injuries.^1^ Achilles tendinopathy (AT) is common in runners with up to 52% of athletes being affected during their lifetime.^2^, ^3^ AT is a clinical diagnosis based on a combination of local Achilles tendon pain, swelling of the Achilles tendon, and an impaired load‐bearing capacity.^4^, ^5^ Treatment outcomes are often disappointing, with persisting symptoms in 35%‐60% of the patients at 5‐year follow‐up.^6^, ^7^

The underlying histopathology of tendinopathy is an increased tenocyte response and local disorganization of the tendon structure.^8^, ^9^ The severity of local tendon tissue disorganization is, however, not directly related to the degree of pain.^10^, ^11^ As a result, peripheral pain is not considered to be the only driver of pain sensation in tendinopathy. Recent evidence found that altered central pain processing might be an important factor in persisting Achilles tendon pain, with pathophysiological pain (central sensitization) as a result.^12^, ^13^, ^14^ These alterations in central pain processing may explain why AT can be resistant to tissue‐based treatment options.^15^

Isometric exercises of several muscle groups (eg, quadriceps muscles) have been found to influence central pain processing.^16^, ^17^ These changes resulted in a substantial immediate analgesic effect in patients with patellar tendinopathy.^18^, ^19^ As a consequence, it has been suggested that isometric exercises can be used to provide immediate pain relief for athletes as “in‐season management.”^20^ To date, only one research group has investigated the immediate analgesic effect of isometric exercises in AT. No reduction in pain was found in this study after the performance of isometric exercises of the calf muscles.^21^ The power of all previous studies was limited, and a clinically relevant immediate effect of exercises cannot be excluded, as no control groups were included in which no exercises were performed.^18^, ^19^, ^21^ As the current treatment advices of tendinopathy are being influenced by these heterogeneous findings,^22^ it is important to clarify the role of isometric exercises in AT.

Our primary aim was to determine the immediate effect of isometric exercises on pain during a functional task in patients with chronic midportion AT. The secondary aim was to compare these results with the effect after the performance of isotonic exercises and rest.

## 2 METHODS

### 2.1 Study design

This quasi‐randomized clinical trial with four intervention arms was conducted as a part of a randomized clinical trial (RCT). The aim of this larger RCT was to evaluate the effect of a high‐volume injection in patients with chronic midportion AT (ClinicalTrials.gov Identifier: NCT02996409). The current study was performed before patients received any type of intervention, and the outcome of the current study did not affect participation in the larger RCT. All patients provided written informed consent prior to participation. The protocol of the study was approved by the regional Medical Ethical Committee (registration number 14‐100). Study findings are reported following the CONSORT guideline, the Pain specific CONSORT supplement checklist, and the TIDieR guideline for reporting interventions. Originally, the aim was to compare three intervention arms: isometric exercises (tiptoes), isotonic exercises, and rest. During the course of the study, there was enhanced insight in this research field that the joint position could influence the analgesic effect as previous research demonstrated that tendon loading varies at different joint positions.^23^ Therefore, we decided to add an intervention arm in which isometric exercises were performed with the ankle dorsiflexed.

### 2.2 Setting and subjects

This clinical trial was performed at the Sports Medicine department in a large district general hospital (Haaglanden Medical Center, Leidschendam, the Netherlands) and a university medical center (Erasmus MC University Medical Center, Rotterdam, the Netherlands). All 80 patients who were included in the RCT participated also in the current study. Additionally, patients with AT who presented after inclusion of the larger RCT was completed were allowed to still participate when visiting one of these research centers. Inclusion was carried out by two sports medicine physicians. Inclusion criteria were as follows: (a) the presence of chronic midportion AT for at least 2 months, (b) no subjective symptomatic improvement after exercise therapy of at least 6 weeks, (c) aged between 18‐70 years, and (d) the presence of Doppler flow on power Doppler ultrasonography. The diagnosis was established based on clinical examination (local tendon pain and swelling of the Achilles tendon 2‐7 cm proximal to its calcaneal insertion, and impaired load‐bearing capacity) by the sports medicine physician. The main exclusion criteria were as follows: (a) a clinical suspicion of other musculoskeletal disorders (insertional AT, inflammatory internal disorders, quinolone‐, corticosteroid‐ or statin‐induced tendinopathy), (b) a previous Achilles tendon rupture or surgery, (c) the inability to perform an exercise program, and (d) a medical condition that could affect the safety of the injection in the larger RCT (eg, peripheral vascular disease or use of anticoagulant medication). Detailed information regarding all eligibility criteria is provided in the trial registration. In case of bilateral symptoms, the patient selected the most symptomatic tendon for inclusion in this study.

### 2.3 Quasi‐randomization

Patients were originally allocated to one of the three intervention arms based on the inclusion number in the larger RCT. Using this method of quasi‐randomization, all patients were equally distributed between the groups. The last 11 participants in the larger RCT and seven extra patients who visited the outpatient department for clinical care (18 in total) were allocated to the additional intervention arm performing isometric exercises with a dorsiflexed position of the ankle.

### 2.4 Interventions

The intervention arms consisted of a specific loading protocol or a period of rest. Patients were told that the loading exercises were performed to determine the maximum amount of weight that could be lifted to personalize the exercise program for the following weeks. Patients in the rest group were asked to sit on a chair while they received verbal instructions regarding the exercise program for the following weeks. The hypothesis of the current study was therefore unknown to all participants. Instructions were provided by a single non‐blinded, trained PhD candidate with 2 years of practical medical training who included patients at both research centers. The interventions were based on previous work in this research field.^18^, ^21^ Of a total of four intervention arms, three groups performed plantar flexor contractions both in seated and standing position and the control group rested. The different loading protocols are presented in Table 1. The duration of all programs was identical (13 minutes). Prior to the programs, patients completed a warming‐up consisting of walking up and down four flights of stairs followed by 1 minute of non‐loaded mobility exercises of the ankle. Patients were subsequently instructed that exercises should be performed at maximal intensity and may be painful. Patients were instructed to ignore pain unless it was unbearable. Patients started with an additional weight of 30 kg for the first set in seated position of the loading protocols. Standardized feedback was provided during the loading protocols (“come‐on, half‐way there”).

**Table 1:** Interventions used in the study

	Joint position (degrees of flexion)	Sets/duration	Recovery (min)	Example of setup in standing position
Isometric (tiptoes)				
Seated	Hip 90°	2 × 45 s	2	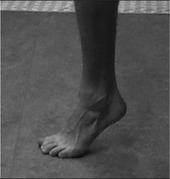
	Knee 90°			
	Ankle 20°			
Standing	Hip 0°	3 × 45 s	2	
	Knee 0°			
	Ankle 20°			
Isometric (dorsiflexed)				
Seated	Hip 90°	2 × 45 s	2	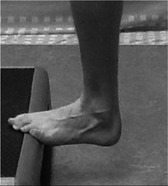
	Knee 90°			
	Ankle −10°			
Standing	Hip 0°	3 × 45 s	2	
	Knee 0°			
	Ankle −10°			
Isotonic				
Seated	Hip 90°	2 × 15 rep	2	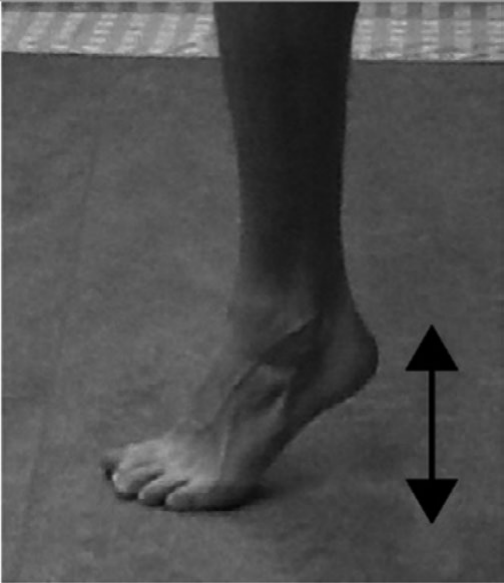
	Knee 90°	(both concentric and eccentric phase 1‐2 s)		
	Ankle 0‐20°			
Standing	Hip 0°	3 × 15 rep	2	
	Knee 0°	(both concentric and eccentric phase 1‐2 s)		
	Ankle 0‐20°			
Rest	—	—	13	

Note

For the plantar flexor contraction groups, five sets of exercises were performed, of which two sessions were performed with the knee bent followed by three sessions with the knee extended. Exercises were performed barefoot under supervision of the researcher. All patients rested for 2 min between sets to allow sufficient recovery. A stopwatch was used to control for the prescribed contraction and rest times. Joint positions of the ankle are presented as positive numbers for plantarflexion and as negative numbers for dorsiflexion.

Abbreviations: rep, repetitions; s, seconds.

The rate of perceived exertion (RPE) was recorded between sets. A score of 0 indicates rest and a score of 10 maximal effort. If exercises were relatively easy to perform (RPE < 7), additional weight was applied using weighted vests up to a maximum of 60 kg and adjustable per 1.5 kg (in seated position resting on the upper leg). To evaluate intensity of the loading protocols, an overall RPE score for the loading protocol was obtained after the performance of all sets of exercises. The weight was lowered in case of unbearable pain or when patients could not complete the contraction times.

### 2.5 Test methods

#### 2.5.1 Demographic details

We recorded the following relevant baseline characteristics: age, sex, body mass index (BMI), activity (active or sedentary), sports participation (hours/wk), affected side, duration of symptoms, and Victorian Institute of Sport Assessment‐Achilles (VISA‐A) score.^24^

#### 2.5.2 Outcome measures

The primary outcome measure we used was the patient‐reported pain after the performance of 10 unilateral hops.^25^, ^26^ We used this outcome measure to test the immediate effect primarily of isometric exercises, but also for isotonic exercises and rest as secondary outcome. According to a recent consensus meeting with patients and healthcare professionals, pain on loading was found to be one of the core domains for tendinopathy.^27^ Patient‐reported pain was measured using a visual analogue scale (VAS) ruler with slider, in which 0 represents no pain and 100 represents the worst pain imaginable. This outcome measure (10 hop VAS score) was assessed at the start of and immediately after the intervention. To evaluate success of the procedure, we evaluated the mean added weight (in kg) during the loading protocol both in the seated and standing position. Additionally, we started to obtain the RPE scores (0‐10) after the first 20 inclusions to determine whether exercises were performed with sufficient effort due to empirical knowledge.

### 2.6 Statistical analyses

Our sample size calculation showed that 16 patients in each group were required to detect a minimal clinically important difference of 20 points on the VAS score (power 0.80, 2‐sided significance level 0.05, SD 20).^21^, ^28^, ^29^ Data were analyzed using SPSS 25.0.0.1 (SPSS Inc.) according to a statistical analysis plan uploaded on clinicaltrials.gov before evaluating the last patient in the RCT. Within‐group differences of the 10 hop VAS scores were analyzed using a Generalized Estimation Equations (GEE) model. To test whether changes in VAS score before and after the program were different between groups, we added the interaction term of intervention arm*pre/post‐testing. Adjustments were made for the following pre‐defined baseline variables: age, sex, BMI, baseline VISA‐A score, and duration of symptoms. Outcomes of the GEE model are presented as estimated marginal means with their 95% confidence interval, unless otherwise stated. Between‐group differences in the RPE score and the added weight during the loading protocol were analyzed using a one‐way ANOVA test. Differences of *P* < .05 were considered to be statistically significant. Missing data were not imputed, but we planned to perform sensitivity analyses if missing data would exceed 5% for a certain time point.^30^

## 3 RESULTS

**Figure 1:**
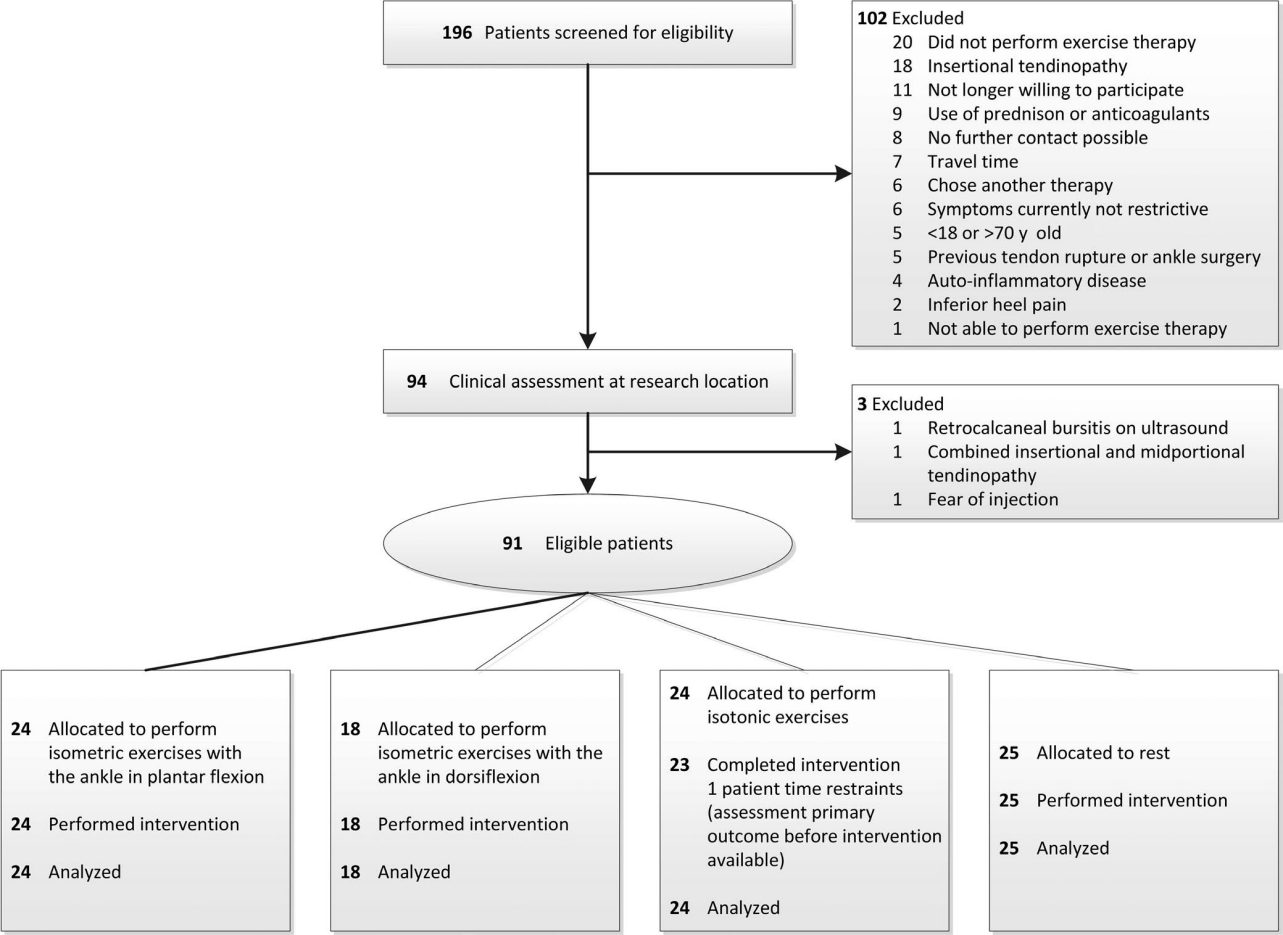
CONSORT flow diagram demonstrating the flow of patients through the study

From December 2016 to August 2019, 196 patients were screened for eligibility and 91 patients were included and allocated to one of the four intervention arms (Figure 1). Only one patient in the isotonic group could not perform the complete loading protocol due to time restraints (assessment primary outcome before intervention available, but not post‐intervention). There were no visual or statistical differences in the majority of the baseline characteristics between the four intervention arms (Table 2), except for the BMI which was higher in the rest group compared to the isometric (tiptoes) group and the VISA‐A score that was lower in the isometric (dorsiflexed) group compared to both the isotonic group and the rest group. Both these characteristics were already included in the GEE model.

**Table 2:** Baseline characteristics of the four intervention arms with between‐group *P*‐values

	Isometric (tiptoes) (n = 24)	Isometric (dorsiflexed) (n = 18)	Isotonic (n = 24)	Rest (n = 25)	*P*‐value^c^
Age, y	47.3 (10.9)	47.6 (9.3)	48.7 (7.4)	49.7 (7.4)	.775
Sex, male, n (%)	9 (38)	9 (50)	13 (54)	14 (56)	.569
BMI	24.5 (4.5)	28.6 (5.4)	26.4 (5.4)	28.5 (5.5)	.026^d^
Activity^a^					
Active, n (%)	22 (92)	10 (56)	19 (79)	20 (80)	.090
Sedentary, n (%)	2 (8)	8 (44)	5 (21)	5 (20)	
Sports participation in desired sport (total hours per week), median (IQR)	4.0 (9.0)	4.3 (3.5)	3.5 (3.5)	3.0 (2.0)	.242
Affected side					
Unilateral, left/right, n (%)	5/10 (62)	3/6 (50)	11/7 (75)	8/7 (60)	.374
Bilateral, n (%)	9 (38)	9 (50)	6 (25)	10 (40)	
Duration of symptoms, wk, median (IQR)	62.0 (120)	104.0 (98)	88.0 (61)	59.0 (46)	.223
VISA‐A score^b^	42.8 (15.1)	32.7 (13.5)	46.0 (15.5)	45.2 (14.7)	.023^e^

Note

Data are presented as mean ± SD unless otherwise specified.

Abbreviations: BMI, body mass index; IQR, interquartile range; n, number of participants; SD, standard deviation; VISA‐A, Victorian Institute of Sports Assessment‐Achilles; wk, weeks; y, years.

^a^ To determine whether participants were active or sedentary we used the ankle‐activity score. If the score was ≥ 4 points, the participant was considered to be active (starting from heavy physical work). If the score was ≤ 3 points, the participant was considered to be sedentary (cycling, equestrian or less activity). Sports participation is only presented for the active group.^33^

^b^ The VISA‐A questionnaire consists of eight questions and covers three domains of Achilles tendon symptoms: pain, activity, and function. Scores vary from 0 to 100 where 100 indicate an asymptomatic person and 0 is defined as maximum pain, no activity, and no function.

^c^ P‐values for between‐group differences in baseline characteristics were calculated using the one‐way ANOVA for normally distributed continuous outcomes, the Kruskal‐Wallis test for non‐normally distributed outcomes, and the chi‐square test for categorical outcomes.

^d^ Post‐hoc testing with Bonferroni correction showed there was a significant difference in BMI between the isometric (tiptoes) group and the rest group (*P* = .05). There were no other significant between‐group differences.

^e^ Post‐hoc testing with Bonferroni correction showed there were significant differences in VISA‐A score between the isometric (dorsiflexed) group and both the isotonic group and the rest group (*P* = .031 and *P* = .047). There were no other significant between‐group differences.

### 3.1 Primary outcome measure—patient‐reported pain

**Figure 2:**
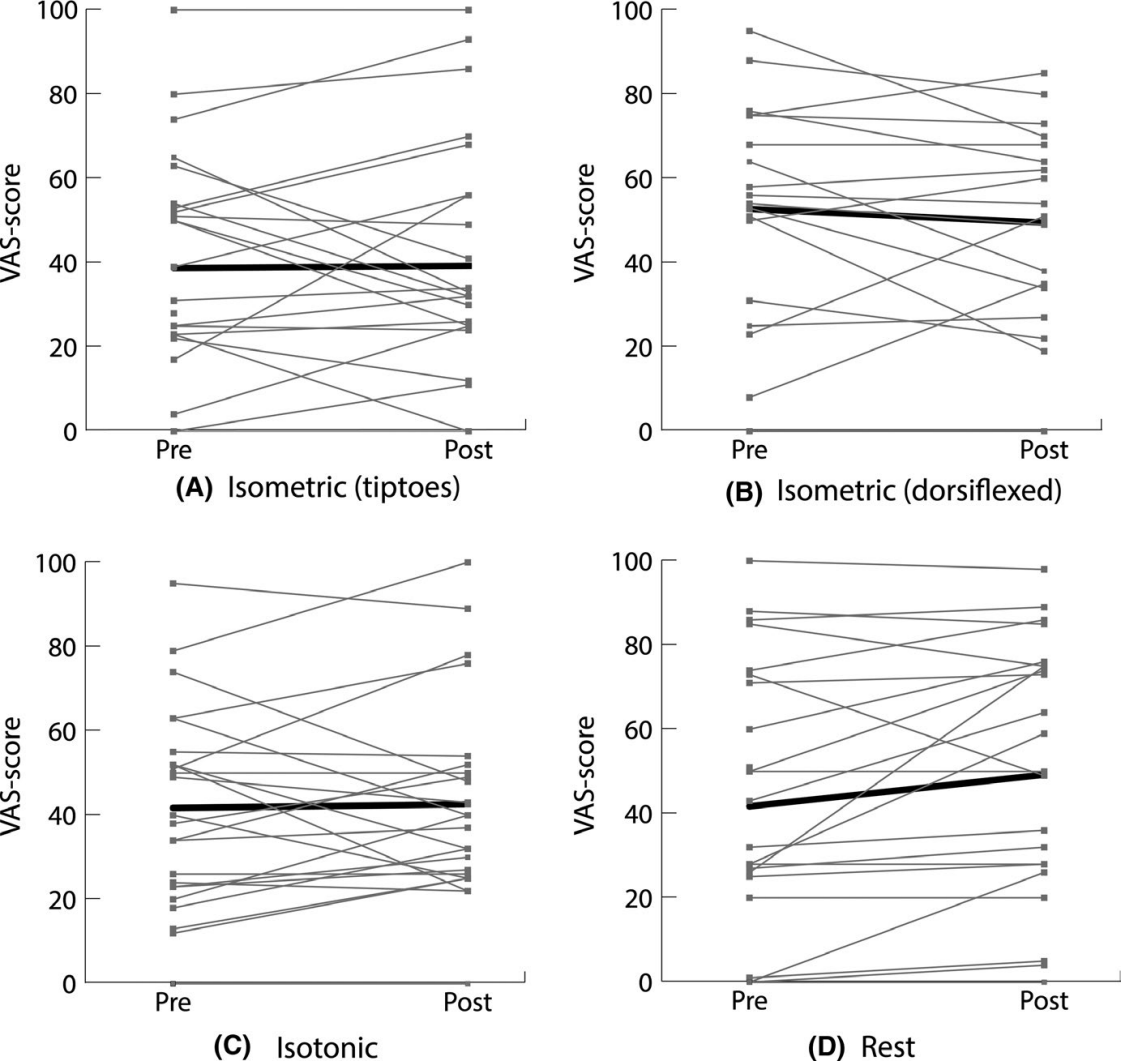
Individual patient data and means per intervention arm (A‐D) for the patient‐reported pain (visual analogue scale [VAS] score) immediately after performing 10 unilateral hops. VAS scores were assessed before (pre) and immediately after the intervention (post). The gray lines represent the individual VAS scores and the black line the raw mean VAS scores of the intervention arm. No statistically significant differences between the four intervention arms were found. A clinical relevant immediate analgesic effect of 20 points was detected in six patients (25%) in the isometric (tiptoes) group, 3 patients (17%) in the isometric (dorsiflexed) group, four patients (17%) in the isotonic group, and 1 patient (4%) rest group

The within‐group differences in estimated mean 10 hop VAS score were not statistically significant in all the intervention groups as presented in Table 3. Ten hop VAS scores are presented as raw individual patient data and mean scores in Figure 2. The interaction term intervention arm*pre/post‐testing was not statistically significant (*P* = .26), meaning that the change in 10 hop VAS score did not differ between the four groups. Between‐group differences were thus all not statistically significant after the interventions (Table 3). The unadjusted analyses are reported in web Appendix 1.

**Table 3:** Outcomes of the generalized estimation equations (GEE) model used to evaluate whether any of the loading protocols/rest provided an immediate analgesic effect

Estimated mean 10 hop VAS scores (0‐100) before and after the performance of one of the interventions				
	Before	After	Within‐group difference	
Isometric (tiptoes)	39.7 (31.8 to 47.6)	39.9 (29.9 to 49.8)	0.2 (−11.2 to 11.5)	
Isometric (dorsiflexed)	41.7 (29.0 to 54.4)	39.8 (28.2 to 51.3)	−1.9 (−13.6 to 9.7)	
Isotonic	44.8 (37.7 to 51.8)	46.2 (37.9 to 54.5)	1.4 (−8.3 to 11.1)	
Rest	44.7 (35.7 to 53.7)	51.9 (43.1 to 60.6)	7.2 (−2.4 to 16.7)	

Between‐group differences immediately after the performance of the loading protocol/rest				
	Isometric (tiptoes)	Isometric (dorsiflexed)	Isotonic	Rest
Isometric (tiptoes)		0.1 (−24.9 to 25.1)	−6.3 (−27.4 to 14.7)	−12.0 (−33.2 to 9.2)
Isometric (dorsiflexed)			−6.4 (−29.9 to 17.0)	−12.1 (−34.9 to 10.7)
Isotonic				−5.7 (−24.8 to 13.5)
Rest				

Note

Adjustments were made for the following pre‐defined baseline variables: age, sex, BMI, baseline VISA‐A score, and duration of symptoms. Outcomes of the GEE model are presented as estimated marginal means with their 95% confidence interval. A higher 10 hop VAS score indicates more pain. Positive values for the between‐group differences correspond to more improvement in 10 hop VAS score compared to the other intervention group. Negative values correspond with less improvement in 10 hop VAS score compared to the other intervention group.

Abbreviation: VAS, visual analogue scale.

### 3.2 Success of procedures

The mean (SD) RPE (0‐10) during the loading protocol was 7.0 (1.7) in the isometric (tiptoes) group (n = 17), 7.7 (1.2) in the isometric (dorsiflexed) group (n = 18), and 7.8 (1.5) in the isotonic group (n = 17). The mean (SD) added weight during the loading protocol in seated position was 28 kg (7) in the isometric (tiptoes) group, 31 kg (4) in the isometric (dorsiflexed) group, and 30 kg (4) in the isotonic group. In the standing position, the mean (SD) added weight was 25 kg (12) in the isometric (tiptoes) group, 16 kg (16) in the isometric (dorsiflexed) group, and 22 kg (12) in the isotonic group. Between‐group differences were not statistically significant for the mean RPE (*P* = .22), the mean added weight in seated position (*P* = .28), and the mean added weight in standing position (*P* = .10).

## 4 DISCUSSION

### 4.1 Summary of main findings

Our trial compared the effects of two different isometric exercises, isotonic exercises, or resting on pain during a functional test in chronic midportion Achilles tendinopathy. Neither isometric nor isotonic exercises provided an immediate analgesic effect.

### 4.2 Clinical implications

Our findings are important and clinically relevant, since the performance of isometric exercises has become increasingly popular as initial treatment and for immediate pain relief in several lower‐limb tendinopathies. The popularity is based on a study that found a large and meaningful decrease in pain score of 6.8 (scale 0‐10) following isometric exercises in patients with patellar tendinopathy, compared to a decrease of 2.6 points following isotonic exercises.^18^ Despite the fact that this was a single study with a very small sample size (n = 6), isometric exercises were implemented rapidly.^20^ Results were quickly extrapolated to other lower‐limb tendinopathies such as AT.^20^

To date, one other research group investigated the analgesic effect of isometric exercises in a relatively small group of patients (n = 16) with chronic midportion AT. Heterogeneous individual responses were found, with no overall meaningful change in pain scores during a functional task after performing isometric exercises.^21^ A difference between that particular study and ours is that in the previous study patients performed exercises using a Wii platform. We performed the exercises in the way they are performed in the clinical setting during rehabilitation with the use of additional weight. Comparable results with no meaningful change were also found in patients with lateral elbow tendinopathy, plantar fasciopathy, and a recent second study in patellar tendinopathy.^19^, ^29^, ^31^ We also demonstrated no meaningful change in pain score after the performance of isometric exercises with either a dorsiflexed or a plantar flexed position of the ankle.

In previous research, isotonic exercises provided immediate pain relief with small magnitude in patellar tendinopathy.^18^ This is the first study to investigate the possible analgesic effect of isotonic exercises in AT. We also found no meaningful change in pain score after isotonic exercises. Our study was the first to include a rest group in which no exercises were performed. Results of the intervention arms performing a loading protocol did not differ from the rest group, indicating that both isometric and isotonic calf‐muscle exercises have no immediate analgesic effect.

### 4.3 Research implications

The previous study in patients with chronic midportion AT demonstrated that the severity of symptoms could play a role in the analgesic effect of isometric exercises. Individuals with higher pain scores worsened in that study, compared to individuals with lower pain scores who improved after isometric exercises.^21^ We did not replicate this finding with our study, as the individual responses to the intervention were very heterogeneous (Figure 2). More research would be needed to determine whether subgroups are present and if so, whether different treatment regimens should be provided. This would involve very large study numbers. We did not investigate the role of isometric or isotonic exercises as an actual treatment for AT, and more research regarding the efficacy of the different exercise programs on the intermediate and long‐term effects is necessary.^32^ Our study shows that both types of exercises also do not aggravate immediate pain. Both exercises are well‐tolerated and could represent a starting point for therapy, self‐efficacy, and self‐management. We also did not investigate the analgesic effect of isometric exercises in patients with a short symptom duration (reactive stage). It could be hypothesized that possible cortical reorganization depends on the chronicity of symptoms, making patients with a chronic symptoms less sensitive to an analgesic effect of isometric exercises.

### 4.4 Strengths and limitations

This is the largest study to date investigating the immediate analgesic effect of both isometric (two groups with different ankle positions) and isotonic exercises in patients suffering from tendinopathy. This is also the first study to include a control group who rested to rule out an analgesic effect from activation of the musculotendinous unit.

Despite our robust research design, there are some methodological limitations. First, we estimated our sample size on a SD of 20 points. However, it transpired that the SD of the changes in the VAS scores was approximately 26 points and thus 28 patients per group would have been required to detect a meaningful change. Additionally, the secondary outcomes could also be slightly underpowered as multiple testing was performed. If we would adjust the alpha level using the Bonferroni method for a total of 6 comparisons, 41 patients per group would have been required. However, if we overlook the mean between‐group differences and the very few responders, it is unlikely that this study does involve a type II error. Furthermore, the abovementioned potential limitations do not influence our primary aim to determine whether isometric exercises provide an immediate analgesic effect. Second, the method of quasi‐randomization was used to allocate patients to the intervention arms. Although this method is not preferable, it was most appropriate as patients were already being randomized to evaluate the effect of an injection treatment. We also adjusted for relevant pre‐defined baseline variables in the GEE model, making it unlikely that differences in baseline characteristics will have influenced results. Third, it was not feasible to blind the outcome assessor (patients) for the type of intervention. However, we did not mention the hypothesis of the study and in doing so avoided influencing patient beliefs regarding the immediate effect of exercise therapy. Fourth, patient beliefs regarding exercise therapy could already be influenced since absence of symptomatic improvement after exercise therapy at short term (at least 6 weeks) was an inclusion criterion. It is, therefore, questionable whether we could extrapolate these results to the broader population of patients with midportion Achilles tendinopathy. Fifth, we did not obtain the RPE scores from the first 20 inclusions. The RPE was still obtained in the majority of the patients (17/24) in both the isometric (tiptoes) group and the isotonic group and in all 18 patients within the isometric (dorsiflexed) group. Sixth, some patients (n = 9) had a 10 hop VAS score of zero at the start of the exercise therapy and could thus not show an immediate analgesic effect. Additional sensitivity analyses excluding those patients did not affect outcomes of this study. Future studies are advised to exclude these patients in their trial.

## 5 PERSPECTIVE

Isometric and isotonic exercises do not result in immediate pain relief in patients with chronic midportion AT. After one small study investigating patients with patellar tendinopathy demonstrated that isometric exercises resulted in a large immediate pain relief, these exercises gained a lot of attention and were implemented rapidly. A recent smaller study in patients with AT was not able to replicate these findings. As a result, there was conflicting evidence for the analgesic effect of isometric exercises. Based on the findings in this study, we do not recommend the use of isometric exercises for immediate pain relief in patients with chronic midportion AT. Future research should focus on the intermediate and long‐term efficacy of isometric and isotonic exercises as a treatment for AT and the analgesic effect of isometric exercises in the reactive stage of AT.

## CONFLICT OF INTEREST

None declared.

## AUTHOR CONTRIBUTIONS

AvdV, JV, PvV, RdV, and RvO involved in study conception/design. AvdV involved in data acquisition. AvdV and RdV analyzed and interpreted the data. AvdV, JV, PvV, RdV, and RvO drafted and critically revised the manuscript. AvdV, JV, PvV, RdV, and RvO gave final approval of the manuscript.

## ETHICAL APPROVAL

Ethics approval was provided by Regional Medical Ethical Committee, registration number 14‐100.

## Supporting information

Appendix S1

## Data Availability

The data that support the findings of this study are available from the corresponding author [AvdV], upon reasonable request.
