# Supplementary material for: Isometric exercises do not provide immediate pain relief in Achilles tendinopathy: A quasi‐randomized clinical trial
Source: Scand J Med Sci Sports. 2020 Jun 14;30(9):1712–21. doi: 10.1111/sms.13728 (PMC7496962; doi:10.1111/sms.13728)
Supplement: Supplementary file 1 — Appendix S1 [file SMS-30-1712-s001.docx]

| Web appendix 1. Outcomes of the Generalized Estimation Equations (GEE) model without adjustment for baseline variables to evaluate whether any of the loading protocols/rest provided an immediate analgesic effect. Outcomes of the GEE-model are presented as estimated marginal means with their 95% confidence interval. A higher 10 hop VAS-score indicates more pain. Positive values for the between-group differences correspond to more improvement in 10 hop VAS-score compared to the other intervention group. Negative values correspond with less improvement in 10 hop VAS-score compared to the other intervention group. | | | | |
| --- | --- | --- | --- | --- |
| Estimated mean 10 hop VAS-scores (0-100) before and after the performance of one of the interventions | | | | |
|  | **Before** | **After** | **Within-group difference** |  |
| Isometric (tiptoes) | 38.7 (28.2 to 49.2) | 38.9 (27.6 to 50.1) | 0.1 (-11.2 to 11.5) |  |
| Isometric (dorsiflexed) | 52.8 (40.9 to 64.7) | 49.5 (39.0 to 60.0) | -3.3 (-15.0 to 9.7) |  |
| Isotonic | 41.2 (32.0 to 50.4) | 42.6 (33.4 to 51.8) | 1.4 (-8.3 to 11.1) |  |
| Rest | 41.7 (29.7 to 53.7) | 48.9 (37.1 to 60.8) | 7.2 (-2.4 to 16.8) |  |
|  |  |  |  |  |
| Between-group differences immediately after the performance of the loading protocol/rest | | | | |
|  | **Isometric (tiptoes)** | **Isometric (dorsiflexed)** | **Isotonic** | **Rest** |
| Isometric (tiptoes) |  | -10.7 (-35.2 to 13.9) | -3.7 (-26.9 to 19.5) | -10.1 (-36.2 to 16.0) |
| Isometric (dorsiflexed) |  |  | 6.9 (-15.3 to 29.1) | 0.6 (-24.6 to 25.8) |
| Isotonic |  |  |  | -6.4 (-30.3 to 17.6) |
| Rest |  |  |  |  |
| Abbreviations: VAS, visual analogue scale. | | | | |
